# Long-course neoadjuvant chemoradiotherapy with versus without a concomitant boost in locally advanced rectal cancer: a randomized, multicenter, phase II trial (FDRT-002)

**DOI:** 10.1186/s13014-019-1420-z

**Published:** 2019-11-29

**Authors:** Jingwen Wang, Yun Guan, Weilie Gu, Senxiang Yan, Juying Zhou, Dan Huang, Tong Tong, Chao Li, Sanjun Cai, Zhen Zhang, Ji Zhu

**Affiliations:** 1 Department of Oncology, Shanghai Medical College, Fudan University, Shanghai, 200032 China; 2 Department of Radiation Oncology, Fudan University Shanghai Cancer Center, Shanghai, 200032 China; 3 Cyberknife Center, Department of Neurosurgery, Huashan Hospital, Fudan University, Shanghai, 200040 China; 4 Neursurgical Institute of Fudan University, Shanghai, 200040 China; 5 Department of Colorectal Surgery, Fudan University Shanghai Cancer Center, Shanghai, 200032 China; 6 Department of Radiation Oncology, The First Affiliated Hospital, College of Medicine, Zhejiang University, Hangzhou, China; 7 Department of Radiation Oncology, 1st Affiliated Hospital of Suzhou (Soochow) University, Suzhou, China; 8 Department of Pathology, Fudan University Shanghai Cancer Center, Shanghai, 200032 China; 9 Department of Radiology, Fudan University Shanghai Cancer Center, Shanghai, 200032 China; 10 Department of Radiation oncology, Huashan Hospital, Fudan University, Shanghai, 200040 China; 11 Department of Cyberknife Center, Huashan Hospital, Fudan University, Shanghai, 200040 China

**Keywords:** Rectal cancer, Neoadjuvant chemoradiotherapy, Intensity-modulated radiation therapy

## Abstract

**Background:**

This study was designed to explore whether an intensified chemoradiotherapy (CRT) led to a better clinical outcome in locally advanced rectal cancer.

**Methods:**

Patients with stage II/III rectal cancer were randomly allocated to receive either pelvic intensity-modulated radiation therapy (IMRT) of 50 Gy/25Fx concurrently with capecitabine and oxaliplatin (Arm A), or pelvic radiation of 50 Gy/25Fx with a concomitant boost of 5 Gy to the primary lesion, followed by a cycle of XELOX 2 weeks after the end of CRT (Arm B). All patients were planned to receive a definitive operation 8 weeks after the completion of CRT and a total of six perioperative chemotherapy cycles of capecitabine and oxaliplatin regardless of pathological result. Pathological complete response (ypCR) was the primary endpoint.

**Results:**

From February 2010 to December 2011, 120 patients from three centers were enrolled in this study. Ninety-five percent patients completed a full-dose chemoradiotherapy as planning. Then 53 and 57 patients received a radical surgery, and 8 and 14 cases were confirmed as ypCR in two groups (*P* = 0.157). The other 10 patients failed to receive a definitive resection because of unresectable disease. Similar toxicities were observed between two groups and more incision healing delay were found in Arm B (3 vs.13, *P* = 0.011). No statistical differences were observed in local-regional control (*P* = 0.856), disease-free survival (*P* = 0.349) and overall survival (*P* = 0.553). Mesorectal fascia (MRF) involvement was an independent prognostic factor for survival in multivariate analysis.

**Conclusions:**

A concomitant boost to oxalipatin-combined preoperative chemoradiotherapy demonstrated a slightly higher pCR rate but delayed incision healing after surgery. The impact of MRF involvement on survival merits further investigations.

**Trial registration:**

NCT01064999 (ClinicalTrials.gov).

## Introduction

With report of results from a series of large randomized clinical trials comparing neoadjuvant pelvic radiotherapy (RT) alone versus RT plus concurrent 5-fluorouracil (5-FU), the standard modality for stage II/III rectal cancer has shifted to preoperative chemoradiotherapy (CRT), followed by total mesorectal excision (TME) and postoperative chemotherapy, which leads to preservation of normal tissue, improvement of tumor regression and excellent local control [1].

The German CAO/ARO/AIO-94 study is the milestone of preoperative CRT [2]. Patients who received preoperative RT in the study had superior local control and reduced toxicity compared with patients in the postoperative group. Subsequently, the EORTC 22921 trial [3] and FFCD9203 trial [4] suggested 5-FU-based CRT resulted in a lower local recurrence rate compared with long-course RT alone, in spite of similar long-term survival. Furthermore, in EORTC 22921 trial, the ypT0–2 group had a significant better OS and DFS than the ypT3–4 group [5]. Similar results were also observed in MDACC’s retrospective study, where tumor regression could be converted to a better long-term prognosis [6]. Moreover, in a pooled analysis [7], it was reported that patients with pCR after CRT have better long-term outcome than do those without pCR (5-year crude DFS: 83∙3% vs 65∙6%). Therefore, in some views, tumor downstaging, especially pCR, was regarded as a goal in neoadjuvant treatment of locally advanced rectal cancer, which motivated the combination of systemic chemotherapy and advanced RT technique in this approaches.

In our center, previous phase II studies reported that oxaliplatin plus 5-FU-based CRT showed good tumor responses and tolerable toxicities [8–10]. Higher radiation dose was reported to contribute to tumor downstaging in literatures [11–13]. Therefore, we designed this randomized trial to explore whether a combined regimen of oxaliplatin-added chemoradiotherapy and a concomitant boost to the primary tumor would further lead to a better clinical outcome.

## Patients and methods

### Patient eligibility

The main inclusion criteria of this study were as follows: (1) newly diagnosed rectal adenocarcinoma; (2) aged between 18 and 75 years old; (3) tumor located within 12 cm from anal verge; (4) clinically staged T3–4 and/or N+; (5) no evidence of distant metastases; (6) had Karnofsky Performance Status score of 60 or more; and (7) had adequate hematologic, renal and hepatic function.

Patients were excluded if they had history of malignant tumor but not including cured skin cancer or cervical cancer in situ, had inflammatory bowel disease, ischemic heart disease, peripheral neuropathy, or psychological disorders. Informed consent must be obtained from every patient before randomization. The procedure of random assignment was performed centrally at the Fudan University Shanghai Cancer Center.

### Treatment schedule

The IMRT technique and tumor volumes definition has been described in our previous trial [9]. For Arm A, the planning dose to the PTV2 (pelvis) were 50 Gy in 25 fractions, five times per week (Monday through Friday) over 5 weeks. For Arm B, the planning doses to the PTV1 (primary tumor) and PTV2 were 55 Gy and 50 Gy in 25 fractions. Electronic portal imaging device (EPID) films were used to verify the isocenter and positioning of each patient for the anterior and lateral gantry positions.

**Fig. 1:**
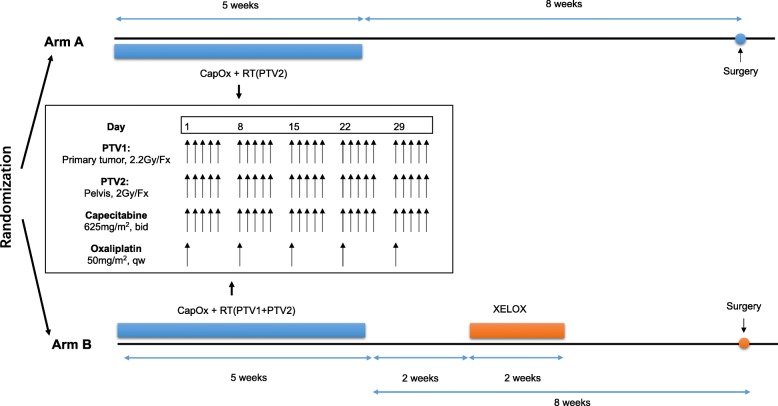
Treatment schedules

Capecitabine and oxaliplatin were given in combination with pelvic radiotherapy. The concurrent chemotherapy regimen was same in both Arms. Capecitabine of 625 mg/m^2^ was given twice daily from Monday to Friday, and oxaliplatin was administered at a fix dose of 50 mg/m^2^/week throughout the entire course of CRT. Two weeks after the end of CRT, one additional cycle of XELOX (capecitabine 1000 mg/m^2^ twice daily on day 1–14 and oxaliplatin 130 mg/m^2^ on day 1) was scheduled for patients in Arm B (Fig. 1).

After the completion of CRT, patients were planned to undergo TME 8 weeks later, while the operation type, such as abdominal-perineal resection (APR) or low anterior resection (LAR), and whether to perform a temporary colostomy, were left to the surgeon to decide. All patients were recommended to receive a total of six cycles of XELOX in perioperative period, regardless of their pathological features.

### Treatment evaluation and follow-up

Baseline evaluation included a comprehensive medical history collection, digital examination, colonoscopy and biopsy, computed tomography (CT) scanning of chest and abdomen, and magnetic resonance imaging (MRI) of pelvis. Endorectal ultrasound (EUS) and positron emission tomography (PET) were not routinely recommended.

Pathologic examination of surgical specimens was performed in accordance with the American Joint Committee on Cancer (AJCC, Version 7) [14]. If less than 12 lymph nodes were found during standard examination for resected lymph nodes, two pathologists needed to double check to verify the number of lymph nodes. The tumor regression grade (TRG) after chemoradiotherapy was recorded according to the AJCC TRG system. TRG 0: Complete tumor response-no viable cancer cells; TRG 1: Moderate response-single or small groups of tumor cells; TRG 2: Minimal response-residual cancer outgrown by fibrosis; TRG 3: Poor response-minimal or no tumor cells killed. PCR was defined as the absence of viable tumor cells in the resection specimens including the primary tumor and lymph nodes (ypT0N0), and near-pCR was defined as ypT0N1a or TRG1 in our study. Those with a margin of circumferential rectal margin (CRM) < 1 mm were marked in positive status [15].

Acute toxicities were defined as toxicities occurred during the entire course of CRT and were evaluated weekly according to the National Cancer Institute Common Toxicity Criteria (CTCAE 4.0). Follow-up was scheduled every 3 months during the first 2 years after surgery, and then every 6 months over the next 3 years. After 5 years, the frequency of follow-up was extended to once each year.

### Endpoints and statistics

An intention-to-treat (ITT) dataset including all eligible patients were used in the analysis procedure. The hypothesis was to increase the ypCR rate from 10% in the Arm A to 25% in the Arm B. A total of 120 patients were required to detect such a difference, with α = 0.20 (two tailed) and power = 0.80. Secondary endpoints were listed as follows: toxicities, sphincter preservation rate, local failure (LF), disease-free survival (DFS) and overall survival (OS).

All features were listed by mean and standard deviations for normal distributional data, by median and interquartile range (IQR) for non-normal distributional data, and by frequency for categorical variables. Comparisons between two groups were performed using χ^2^ tests for categorical variable. Survival curves were estimated using the Kaplan-Meier method and compared with Log-rank test. Cox proportional hazards regression was used for univariate and multivariate modeling and for examining the prognostic significance of the variables identified in the model. *P* values of less than 0.05 were taken to indicate statistically significant differences.

## Results

### Baseline characteristics

**Fig. 2:**
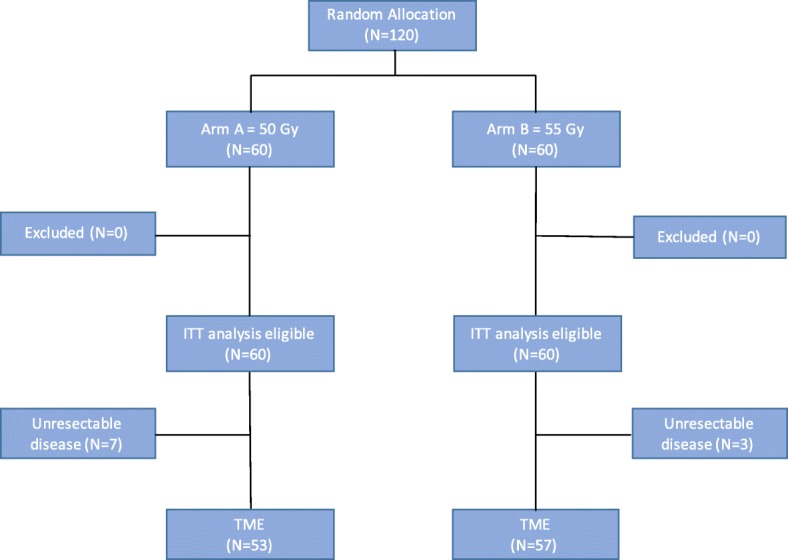
CONSORT for FDRT-002 trial. Arm A, 5 weeks of treatment with radiotherapy 50 Gy/25 fractions with concurrent capecitabine 625 mg/m^2^ twice daily 5 days per week and oxaliplatin 50 mg/m^2^ once weekly; Arm B, 5 weeks of treatment with radiotherapy 55 Gy/25 fractions with concurrent capecitabine 625 mg/m^2^ twice daily 5 days per week and oxaliplatin 50 mg/m^2^ once weekly, followed by a cycle of XELOX. ITT, intention to treat; TME, total mesorectal excision

From February 2010 to December 2011, 120 eligible patients in three centers (Fudan University Shanghai Cancer Center, Shanghai, China; The First Affiliated Hospital, College of Medicine, Zhejiang University, Hangzhou, China; The First Affiliated Hospital of Suzhou University, Suzhou, China) were randomly assigned in the trial, and the final analysis was performed in ITT set including all 120 cases (Fig. 2).

Patient characteristics were presented in Table 1. Eighty-four were male and 36 were female, and the median age was 56 (range 22–75). More cases were diagnosed with cT3, cN+, negative mesorectal fascia (MRF) and with the distance from anal verge less than 5 cm. No statistical differences were observed between two groups in the baseline characteristics (Table 1).

**Table 1:** Baseline characteristics of patients (*N* = 120)

	Arm A		Arm B		Total		*P* value
	No.	%	No.	%	No.	%	
Gender							
Male	42	70.00%	42	70.00%	84	70.00%	
Female	18	30.00%	18	30.00%	36	30.00%	1.000
Age, years							
< =55	27	45.00%	27	45.00%	54	45.00%	
> 55	33	55.00%	33	55.00%	66	55.00%	1.000
Clinical T stage							
T3	40	66.70%	45	75.00%	85	70.80%	
T4	20	33.30%	15	25.00%	35	29.20%	0.315
Clinical N stage							
N0	14	23.30%	14	23.30%	28	23.30%	
N+	46	76.70%	46	76.70%	92	76.70%	1.000
MRF							
-	36	60.00%	38	63.30%	74	61.70%	
+	24	40.00%	22	36.70%	46	38.30%	0.707
Location from anal verge, cm							
< =5 cm	40	66.70%	40	66.70%	80	66.70%	
> 5 cm	20	33.30%	20	33.30%	40	33.30%	1.000
Length of the tumor							
< =5 cm	31	51.70%	22	36.70%	53	44.20%	
> 5 cm	29	48.30%	38	63.30%	67	55.80%	0.098
Total	60	50%	60	50%	120	100%	

Arm A, 5 weeks of treatment with radiotherapy 50 Gy/25 fractions with concurrent capecitabine 625 mg/m^2^ twice daily 5 days per week and oxaliplatin 50 mg/m^2^ once weekly; Arm B, 5 weeks of treatment with radiotherapy 55 Gy/25 fractions with concurrent capecitabine 625 mg/m^2^ twice daily 5 days per week and oxaliplatin 50 mg/m^2^ once weekly, followed by a cycle of XELOX

*Abbreviations*: *MRF* Mesorectal fascia

### Compliance and toxicities

Full-dose radiotherapy was given in 98.3% patients in both groups. One case in Arm B received single drug during the whole course of CRT for the reason that he refused intravenous chemotherapy. Almost all patients completed five cycles of weekly oxaliplatin, except that one and four patients terminated oxaliplatin in the second and fourth cycles, respectively. No further chemotherapy dose modification was recorded.

The overall acute grade 3–4 toxicities were 18.3 and 25.0% in the two groups, Arm A and Arm B, respectively. Diarrhea, radiation dermatitis and nausea were the three most common toxicities in both groups, though a slightly higher number of above cases were found in Arm B without significant difference (Table 2).

**Table 2:** Acute toxicity according to CTCAE 4.0, on all patients receiving treatment

Adverse Event	Grade 1–2				Grade 3–4				*P* value*
	Arm A (*N* = 60)		Arm B (*N* = 60)		Arm A (*N* = 60)		Arm B (*N* = 60)		
	No.	%	No.	%	No.	%	No.	%	
Diarrhea	21	35.0%	16	26.7%	4	6.7%	6	10.0%	0.721
Nausea	35	58.3%	31	51.7%	2	3.3%	3	5.0%	0.683
Vomiting	5	8.3%	7	11.7%	2	3.3%	2	3.3%	0.608
Anorexia	13	21.7%	18	30.0%	0	0.0%	1	1.7%	0.203
Radiodermatitis	27	45.0%	22	36.7%	8	13.3%	10	16.7%	0.807
Anemia	37	61.7%	34	56.7%	0	0.0%	0	0.0%	0.581
leukopenia	16	26.7%	21	35.0%	2	3.3%	2	3.3%	0.360
Neutropenia	15	25.0%	20	33.3%	2	3.3%	3	5.0%	0.247
Thrombopenia	27	45.0%	25	41.7%	2	3.3%	1	1.7%	0.543
Overall	40	66.7%	43	71.7%	11	18.3%	15	25.0%	0.077

Arm A, 5 weeks of treatment with radiotherapy 50 Gy/25 fractions with concurrent capecitabine 625 mg/m^2^ twice daily 5 days per week and oxaliplatin 50 mg/m^2^ once weekly; Arm B, 5 weeks of treatment with radiotherapy 55 Gy/25 fractions with concurrent capecitabine 625 mg/m^2^ twice daily 5 days per week and oxaliplatin 50 mg/m^2^ once weekly, followed by a cycle of XELOX

*Abbreviations*: *MRF* Mesorectal fascia

*All *P* values are comparison of grade 3 and 4 adverse events between the two arms

### Surgical procedures and pathological outcome

Radical surgery was performed in 53 (88.3%) patients in arm A and 57 (95.0%) in arm B after a median interval between CRT and surgery of 51 and 58 days, respectively. The other ten patients failed to undergo a surgery due to unresectable diseases. Anterior resection procedure was performed in 13 and 22 patients (24.5 and 38.6% of the patients who underwent surgery in arms A and arm B), respectively (Table 3). Arm B demonstrated a slightly better ypCR, ypT and ypN stage. PCR were found in 22 cases, 8 in Arm A and 14 in Arm B. There were 45.3 and 31.6% patients demonstrating positive lymph nodes in two groups, respectively. The rate of anastomotic fistula and low anterior resection syndrome were equally low in both arms, but more delayed incision healing were observed in Arm B (Table 4).

**Table 3:** Pathologic characteristics of the operative specimen from patients (*N* = 110^a^)

	Arm A		Arm B		Total		*P* value
	No.	%	No.	%	No.	%	
Type of surgery							
Miles	36	67.90%	32	56.10%	68	61.80%	
Anterior resection	13	24.50%	22	38.60%	35	31.80%	
Hartmann	4	7.50%	3	5.30%	7	6.40%	0.279
ypT stage							
ypT0	10	18.90%	16	28.10%	26	23.60%	
ypT1–2	16	30.20%	24	42.10%	40	36.40%	
ypT3–4	27	50.90%	17	29.80%	44	40.00%	0.077
ypN stage							
N0	29	54.70%	39	68.40%	68	61.80%	
N+	24	45.30%	18	31.60%	42	38.20%	0.139
pCR							
pCR	8	15.10%	14	24.60%	22	20.00%	
non-pCR	45	84.90%	43	75.40%	88	80.00%	0.215
Total	53		57		110		

Arm A, 5 weeks of treatment with radiotherapy 50 Gy/25 fractions with concurrent capecitabine 625 mg/m^2^ twice daily 5 days per week and oxaliplatin 50 mg/m^2^ once weekly; Arm B, 5 weeks of treatment with radiotherapy 55 Gy/25 fractions with concurrent capecitabine 625 mg/m^2^ twice daily 5 days per week and oxaliplatin 50 mg/m^2^ once weekly, followed by a cycle of XELOX

*Abbreviations*: *pCR* Pathological complete response;^a^Patients who did not undergo surgery excluded

**Table 4:** Postoperative surgical complications (*N* = 110^a^)

Complications	Arm A (*N* = 53)	Arm B (*N* = 57)	*P* value
Total	4	14	0.016
Local fistula	1	0	0.482
LARS^a^	0	1	0.491
Delayed incision healing	3	13	0.011

Arm A, 5 weeks of treatment with radiotherapy 50 Gy/25 fractions with concurrent capecitabine 625 mg/m^2^ twice daily 5 days per week and oxaliplatin 50 mg/m^2^ once weekly; Arm B, 5 weeks of treatment with radiotherapy 55 Gy/25 fractions with concurrent capecitabine 625 mg/m^2^ twice daily 5 days per week and oxaliplatin 50 mg/m^2^ once weekly, followed by a cycle of XELOX

*Abbreviations*: *LARS* Low anterior resection syndrome;^a^Patients who did not undergo surgery excluded

In additional analysis for tumor response, good response was defined as pCR plus near-pCR, the others were marked as poor response, including those who could not receive surgery for unresectable lesion. A higher good response rate was observed in Arm B (30% vs. 55%, *P* = 0.006) (Table 5). Two patients in Arm A received additional postoperative radiotherapy of 10–15 Gy because of a positive margin.

**Table 5:** pCR rate and patient response (*N* = 120^a^)

	Arm A		Arm B		Total		*P* value
	No.	%	No.	%	No.	%	
pCR							
pCR	8	13.3%	14	23.3%	22	18.3%	
non-pCR	52	86.7%	46	76.7%	98	81.7%	.157
Response							
Good	18	30.0%	33	55.0%	51	42.5%	
Poor	42	70.0%	27	45.0%	69	57.5%	.006

Good response: pCR plus near pCR; Poor response: others

*Abbreviations*: *pCR* Pathological complete response

^a^Patients who did not undergo surgery included

### Long-term prognosis

A total of 49 patients in Arm A and 51 in Arm B received adjuvant CT. The most frequent regimen was XELOX, with median of three cycles.

**Fig. 3:**
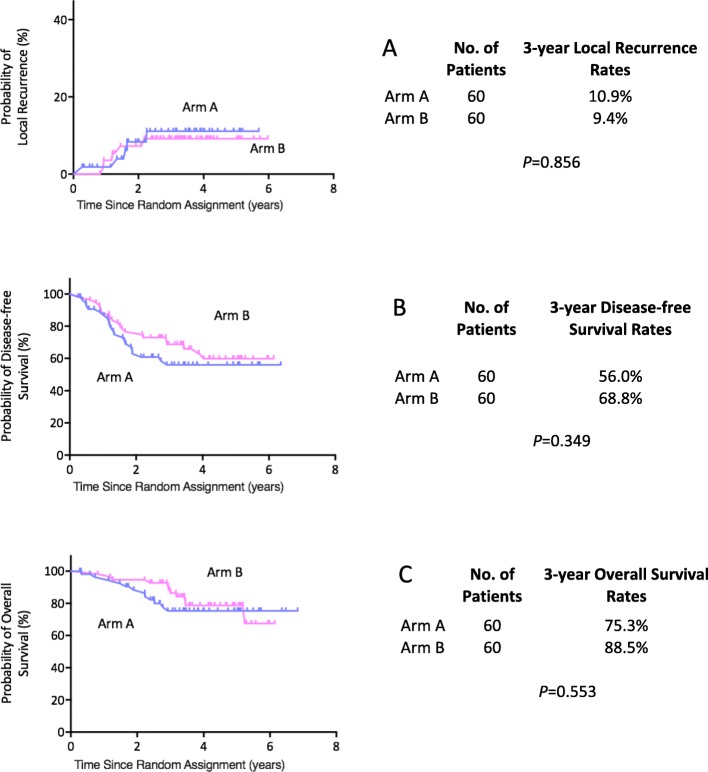
Long-term survival. **a** Cumulative incidence of local recurrences; **b** Disease-free survival and **c** Overall survival in the intention-to-treat population

With a median follow-up of 42 months (range 3.2–80.7 months), ten patients were confirmed with local recurrence and 26 patients were diagnosed with distant metastases. A total of 20 patients died, 17 of cancer-related disease, 3 of other reasons. There were no significant differences in 3-year local-regional failure (10.9% vs. 9.4%, *P* = 0.856), 3-year DFS (56.0% vs. 68.8%, *P* = 0.349) and 3-year OS (75.3% vs. 88.5%, *P* = 0.553) between two groups (Fig. 3).

### Univariate and multivariate analysis of OS, DFS and LC

All potential prognostic factors, including demographic and clinical features were evaluated using Kaplan-Meier and Cox model (Table 6). Positive MRF was associated with poorer local control, DFS and OS; ypN and tumor response were correlated with DFS and OS; pCR was in favor of longer DFS. In the multivariate Cox model, ypT, ypN and pCR were excluded because they exhibited a strong correlation with tumor response. MRF was the only independent prognostic factor for local control, DFS and OS simultaneously. Tumor response was also an independent risk factor for DFS (Table 7).

**Table 6:** Results of the univariate analysis of prognostic factors on overall survival, disease-free survival, local control

Factor	Overall Survival			Disease-free Survival			Local Control		
	HR	95%CI	*P*	HR	95%CI	*P*	HR	95%CI	*P*
Gender									
male / female	0.849	0.334–2.160	0.731	1.280	0.674–2.434	0.451	0.588	0.125–2.771	0.502
Age									
> 55 / <=55	0.737	0.325–1.672	0.465	0.872	0.476–1.599	0.658	2.002	0.518–7.742	0.315
Treatment									
high-dose / low-dose	N/A	N/A	N/A	N/A	N/A	N/A	0.892	0.258–3.082	0.856
cT stage									
cT4 / cT3	1.319	0.542–3.209	0.542	1.166	0.596–2.278	0.654	0.318	0.040–2.511	0.277
cN stage									
cN+ / cN0	1.051	0.390–2.835	0.921	1.036	0.496–2.166	0.925	2.678	0.339–21.146	0.350
MRF									
MRF+ / MRF-	2.971	1.297–6.802	0.010	2.485	1.351–4.572	0.003	4.802	1.240–18.590	0.023
Location									
> 5 cm / ≤5 cm	1.191	0.515–2.753	0.683	0.888	0.462–1.709	0.723	0.484	0.103–2.280	0.359
Length									
> 5 cm / ≤5 cm	1.563	0.662–3.693	0.308	1.219	0.658–2.260	0.528	2.059	0.532–7.965	0.296
ypT									
ypT1–2/ypT0	1.047	0.294–3.734	0.944	1.672	0.641–4.363	0.294	1.797	0.187–17.278	0.612
ypT3–4 / ypT0	1.238	0.372–4.114	0.728	2.116	0.838–5.339	0.113	3.620	0.436–30.080	0.234
ypN									
ypN+ / ypN0	3.518	1.346–9.191	0.010	2.234	1.178–4.234	0.014	0.776	0.201–3.004	0.714
pCR									
non-pCR / pCR	2.728	0.639–11.646	0.175	2.656	0.946–7.459	0.064	2.058	0.261–16.257	0.494
Response									
Poor / Good	2.574	1.013–6.544	0.047	2.228	1.156–4.297	0.017	1.224	0.345–4.339	0.755

Good response: pCR plus near pCR; Poor response: others

*Abbreviations*: *HR* Hazard ratio, *pCR* Pathological complete response, *MRF* Mesorectal fascia

**Table 7:** Results of the multivariate analysis of prognostic factors on overall survival, disease-free survival, local control

Factor	Factor	*P* value	95%CI	HR
Overall Survival	MRF+/MRF-	0.023	4.802–1.240	18.590
Disease-free Survival	MRF+/MRF-	0.011	2.231–1.203	4.136
	Poor response/Good response	0.047	1.959–1.007	3.808
Local Control	MRF+/MRF-	0.010	2.971–1.297	6.802

*Abbreviations*: *HR* Hazard ratio, *MRF* Mesorectal fascia

## Discussion

This phase II randomized trial was designed to compare two different dose-intensified regimens in neoadjuvant therapy. With the preset alpha of 0.20, the primary endpoint reached an expected higher pCR rate (13.3% vs. 23.3%, *P* = 0.157). Furthermore, the intensified dose regimen was associated with better tumor response but more delayed incision healing after surgery in the experimental arm (Arm B). Therefore, our results suggested that the enhanced dose regimen with a concomitant boost to the primary tumor in oxaliplatin-added neoadjuvant chemoradiotherapy warrant more attentions.

In the design of our study, the hypothesis was that the intensified treatment would lead to a better tumor regression, as well as a longer survival. Therefore, enhanced treatment dose intensity was regarded as an effective method, including both radiotherapy and chemotherapy dose. Plenty of early phase II trials indicated that it was beneficial to add oxaliplatin to standard FU-based CRT [16], and several continuous small sample size studies in our center also showed similar results [8, 9]. Therefore, based on literature review at that time, oxaliplatin was administered in both two arms in this trial. Additionally, our previous study reported that 23.7% patients were evaluated as pCR, who received IMRT to the pelvis of 50 Gy and a concomitant boost of 5 Gy to the primary tumor, in combination with oxaliplatin and capecitabine, followed by a cycle of XELOX before surgery [8]. Therefore, a concomitant boost to the primary tumor was regarded as an efficient supplementary to the approach and this regimen was recommended as the experimental arm in this trial.

Noticed the rate of pCR was 23.3% in the experimental arm compared with 13.3% in the controlled arm in the present study, it may be resulted from both chemotherapy and radiation dose escalation. Even though in the next six phase III trials, the mainstream view considered no significant benefit was brought by additional oxaliplatin [17–24], we still hold some different opinions as follows: the completion proportion of oxaliplatin during CRT ranges from 41 to 94.5% in these trials, which may be a leading cause of inconsistent results. It is worthy more attention that two trials having an enhanced pCR rate demonstrated a high completion proportion of oxaliplatin of 85% or more (CAO/ARO/AIO-04 and FORWARC trial) [19, 21]. Therefore, a pooled analysis including all above trials is expected to identify the subgroup population who can really benefit from additional oxaliplatin.

Furthermore, with the development of RT technique, such as IMRT and brachytherapy, higher preoperative pelvic irradiation dose was delivered in some studies. Radiation dose was regarded as a significant factor in the degree of tumor downstaging as reported by Mohiuddin et al. [11], whose study suggested that the rate of pCR was significantly correlated with RT dose, as patients treated to a dose of less than 50 Gy had a downstaging rate of 67% and a pCR rate of 3%, compared with a downstaging rate of 89% and a pCR rate of 45% at doses of more than 55 Gy (*P* = 0.05). What’s more, in The Radiation Oncology Group (RTOG) 0012 phase II trial [12], patients were randomly assigned to either hyper-fractionated pelvic RT plus continuous infusion 5-FU or standard pelvic RT plus continuous infusion 5-FU and irinotecan, and both arms achieved very high pCR rates of 26% in each arm. So, the intensified RT dose plays a role in preoperative treatment of LARC.

In the uni- and multi-variate analysis of our study, we found that MRF status played as independent prognostic factors for long-term prognosis. As reported in the experience of MERCURY trial [25], MRI-defined involvement of CRM is an independent prognostic factor for 5-year overall survival (mrCRM+ vs mrCRM-: 62.2% vs 42.2%), for DFS (67.2% vs 47.3%) and for local recurrence with a hazard ratio of 3.5 (*P* < 0.05). In our study, the results was similar. Nevertheless, since the sample size was small, we couldn’t perform subgroup analysis to verify the conclusion.

However, this study had some limitations. Firstly, the control arm was not the current standard of care for locally advanced rectal cancer. As we stated previously, when we designed and conducted this study, oxaliplatin was still in high expectation in the neoadjuvant CRT phase. Secondly, it was indeed difficult to clarify which is the main reason for a better tumor response and more severe toxicities since radiation dose and consolidation chemotherapy were enhanced simultaneously in the experimental group. Thirdly, we didn’t notice any long-term benefit in the group with higher pCR rate. As it was common in most phase II trials in neoadjuvant treatment of LARC, the improvement of pCR rate was difficult to convert to a longer survival. Last but not the least, this study was originally designed as a two-stage trial with a total sample size of 240. But because of the disappointed results of several phase III trials about additional oxaliplatin in neoadjuvant CRT, we terminated this study after the completion of the first phase.

## Conclusion

A concomitant boost to oxalipatin-combined preoperative chemoradiotherapy demonstrated contribution to tumor regression with acceptable acute toxicity, but led to delayed incision healing after surgery. More data are needed to assess the impact of dose-intensified radiotherapy on long-term survival. The impact of MRF involvement on survival merits further investigation. Considered the experimental regimen was not compared to the standard of care, the conclusion should be interpreted cautiously.

## Data Availability

The datasets used and/or analyzed during the current study are available from the corresponding author on reasonable request.

## Abbreviations

AJCC: American Joint Committee on Cancer
APR: Abdominal-perineal resection
CRM: Circumferential rectal margin
CRT: Chemoradiotherapy
CT: Computed tomography
CTCAE: National Cancer Institute Common Toxicity Criteria
DFS: Disease-free survival
EPID: Electronic portal imaging device
IMRT: Intensity-modulated radiation therapy
ITT: Intention-to-treat
LAR: Low anterior resection
LARC: Locally advanced rectal cancers
LR: Local control rate
MRI: Magnetic resonance imaging
OS: Overall survival
PET: Positron emission tomography
PTV: Planning target volume
TME: Total mesorectal excision
UICC: International Union against Cancer
